# Case Report: A Novel TNFAIP3 Mutation Causing Haploinsufficiency of A20 With a Lupus-Like Phenotype

**DOI:** 10.3389/fimmu.2021.629457

**Published:** 2021-02-19

**Authors:** Zachary R. Shaheen, Sarah J. A. Williams, Bryce A. Binstadt

**Affiliations:** ^1^ Division of Rheumatology, Allergy & Immunology, Department of Pediatrics, University of Minnesota Medical School, Minneapolis, MN, United States; ^2^ Department of Laboratory Medicine and Pathology, University of Minnesota Medical School, Minneapolis, MN, United States

**Keywords:** A20, haploinsufficiency of A20, tumor necrosis factor alpha-induced protein 3, lupus, lymphadenopathy, hypogammaglobulinemia, case report

## Abstract

Genetic mutations that result in loss-of-function of the protein A20 result in an early-onset autoinflammatory disease—haploinsufficiency of A20 (HA20). The reported clinical presentations of HA20 include a Behcet’s disease-like phenotype and a more lupus-like phenotype. We have identified a novel mutation in the gene encoding A20 in a pediatric patient with chronic lymphadenopathy, lupus-like symptoms, and progressive hypogammaglobulinemia. This case illustrates the wide range of clinical symptoms, including immunodeficiency, that can occur in patients with HA20.

## Introduction

The gene *TNFAIP3* (tumor necrosis factor alpha-induced protein 3) encodes the protein A20, an essential negative regulator of NF-κB-mediated inflammation (1, 2). A20 is a potent inhibitor of NF-κB signaling in response to TNF-α, numerous other pro-inflammatory cytokines, and innate immune receptor activation *via* its ubiquitin-editing domains (3, 4). Heterozygous loss-of-function mutations in A20 causes haploinsufficiency of A20 (HA20) and a Behcet’s-like disease in children (prominent mucosal ulcers and intestinal symptoms) (5), and have been associated with a number of other autoimmune and autoinflammatory diseases (5–8) as well as lymphomas and other cancers (7). We present a case of a child with a novel *TNFAIP3* mutation causing HA20, with clinical features resembling systemic lupus erythematosus with associated recurrent lymphadenopathy and progressive hypogammaglobulinemia.

## Case Description, Diagnostic Assessment, Therapeutic Intervention, Follow-Up, and Outcomes

During the patient’s first year of life, she was diagnosed with atopic dermatitis (controlled non-pharmacologically) and had a documented history of viral upper respiratory illness, bacterial acute otitis media, pneumonia, and one hospitalization for sepsis and respiratory symptoms.

At 2.5 years of age, the patient presented with persistent posterior cervical lymphadenopathy, which was monitored and eventually surgically resected at the age of 3; the pathologic findings were non-specific. She returned several times between the ages of 5–7 years for posterior cervical lymphadenopathy and fever of unknown origin in the absence of other symptoms. When she was 7.5 years old, she was hospitalized for concern of meningitis with evolving symptoms of prolonged fevers, headache, and neck pain. Cerebrospinal fluid (CSF) studies were notable for elevated white blood cells (WBCs; 0.8 x 10^9^ cells/L), but tests for infectious diseases including Gram stain, bacterial culture, tick-borne pathogen screening, tuberculosis, and viral testing were all negative. During this hospitalization, she became pancytopenic requiring transfusion (WBC nadir of 3.1 x 10^9^ cells/L, and thrombocytopenia to 30 x 10^9^ cells/L); a bone marrow biopsy demonstrated normal cellularity and no pathologic changes. Repeat biopsy of a right posterior cervical lymph node showed features consistent with atypical lymphoid hyperplasia but not a lymphoproliferative disorder. Rheumatology was consulted during this hospitalization for persistent fevers, emergence of a maculopapular rash, and concern for macrophage activation syndrome. Notable laboratory values at that time included an elevated anti-nuclear antibody (ANA of 12.4, normal <1.0) and anti-double-stranded DNA antibodies (dsDNA of 167, normal <29 IU/ml), negative anti-extractable nuclear antigen (ENA) antibodies (negative SS-A/Ro, SS-B/La, scleroderma, Smith, and ribonucleoprotein antibodies), negative cardiolipin antibodies and negative lupus anticoagulant, positive anti-platelet antibodies (Gp2b3a), normal complement C3 and C4 levels, elevated ferritin (peak of 2300 ng/mL), normal triglycerides, and moderate elevation of creatine kinase (CK) and transaminases. Serum immunoglobulin levels were normal to elevated (IgG 1530, normal 500–1,400 mg/dl; IgA 360, normal 47–249 mg/dl; IgM 188, normal 47–252 mg/dl; IgE 535, normal 0–150 kIU/L); serum protein electrophoresis revealed polyclonal gammopathy. Rheumatology continued to follow this patient in clinic after hospitalization due to a suspicion of an evolving inflammatory illness with intermittent fevers of unclear etiology. Autoimmune lymphoproliferative syndrome (ALPS) was considered, though CD4/CD8 double-negative T-cell (DNT) populations were within normal range: absolute α/β-TCR (T-cell receptor) DNT of 7 (normal <35 cell/µl); percentage α/β-TCR DNT of 0.5% (normal <2.0%). Systemic lupus erythematosus (SLE), Castleman’s disease, and periodic fever syndromes were considered, but no formal diagnosis was established and immune modulating medications were not yet initiated.

After multiple disease flares between the ages of 8 and 11 years which manifested as a combination of discoid malar rash, tender cervical lymphadenopathy, sub-nephrotic range proteinuria, along with serologic evidence of persistently positive ANA, anti-dsDNA, anti-ribosomal P antibodies, suppressed C3, C4, and CH50, the provisional diagnosis of systemic lupus erythematosus (SLE) was made. Hydroxychloroquine (HCQ), mycophenolate mofetil (MMF), prednisone, and rituximab were all gradually added to control disease flares. During these years she also had persistently abnormal lung exams and was diagnosed with bronchiectasis following imaging, bronchoalveolar lavage, and a negative infectious diseases work-up. At age 11, she was hospitalized for dehydration and neutropenic fevers with an absolute neutrophil count (ANC) of 0 x 10^9^ cells/L, anemia, and an inappropriately low reticulocyte count. Her fevers and neutropenia resolved after receiving broad spectrum antibiotics and discontinuing the MMF.

**Figure 1:**
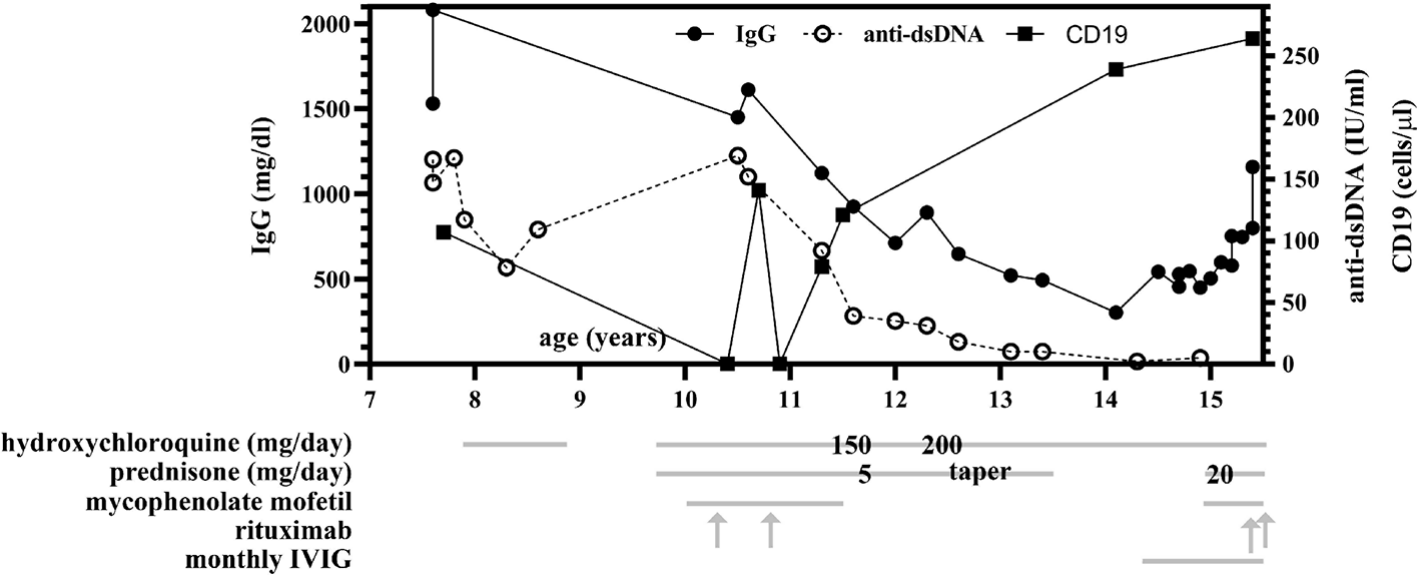
Total IgG and anti-dsDNA levels over time and compared to treatment regimen. Between ages 11 and 14, progressive hypogammaglobulinemia was observed and persisted despite discontinuation of mycophenolate mofetil (MMF) and rituximab infusions, with normalization of IgG levels at age 15 following monthly intravenous immunoglobulin (IVIG) infusions. Anti-dsDNA antibody levels decreased to within normal range (<10 IU/ml) following immunosuppressive therapy initiation at age 10. Treatment with hydroxychloroquine (HCQ), prednisone, MMF, rituximab, and IVIG are indicated by grey lines, and when specific dosing was identified by chart review, is additionally overlaid on the gray line at the time of therapy initiation or dosing change. MMF is twice daily dosing (BID) and was initiated at 500 mg per dose and increased to 1,000 mg twice daily at age 15. At age 10, she received two doses of 500 mg rituximab; at age 15, she received two doses of 1000 mg rituximab.

Following that hospitalization, HCQ and prednisone were continued. Over the course of the next 3 years (ages 12–14), her disease process was well controlled, and oral prednisone was gradually discontinued. During this time, her complement C3 and C4 levels were normal or elevated and anti-dsDNA antibody titers decreased, but she had progressively worsening hypogammaglobulinemia (**Figure 1**). She began developing frequent urinary tract infections (UTIs), ear infections, and atypical pneumonia, along with cough, fevers, and conductive hearing loss with magnetic resonance imaging (MRI) findings of sinus effusions. Monthly intravenous immunoglobulin (IVIG) replacement was initiated, and her underlying diagnosis was reconsidered. Notably, repeat autoantibody testing for other connective tissue diseases, including RNP, Smith, SS-A/Ro, SS-B/La, Scleroderma, cardiolipin, as well as lupus anti-coagulant, remained negative (tested 7 times in total over ages 7-15).

**Figure 2:**
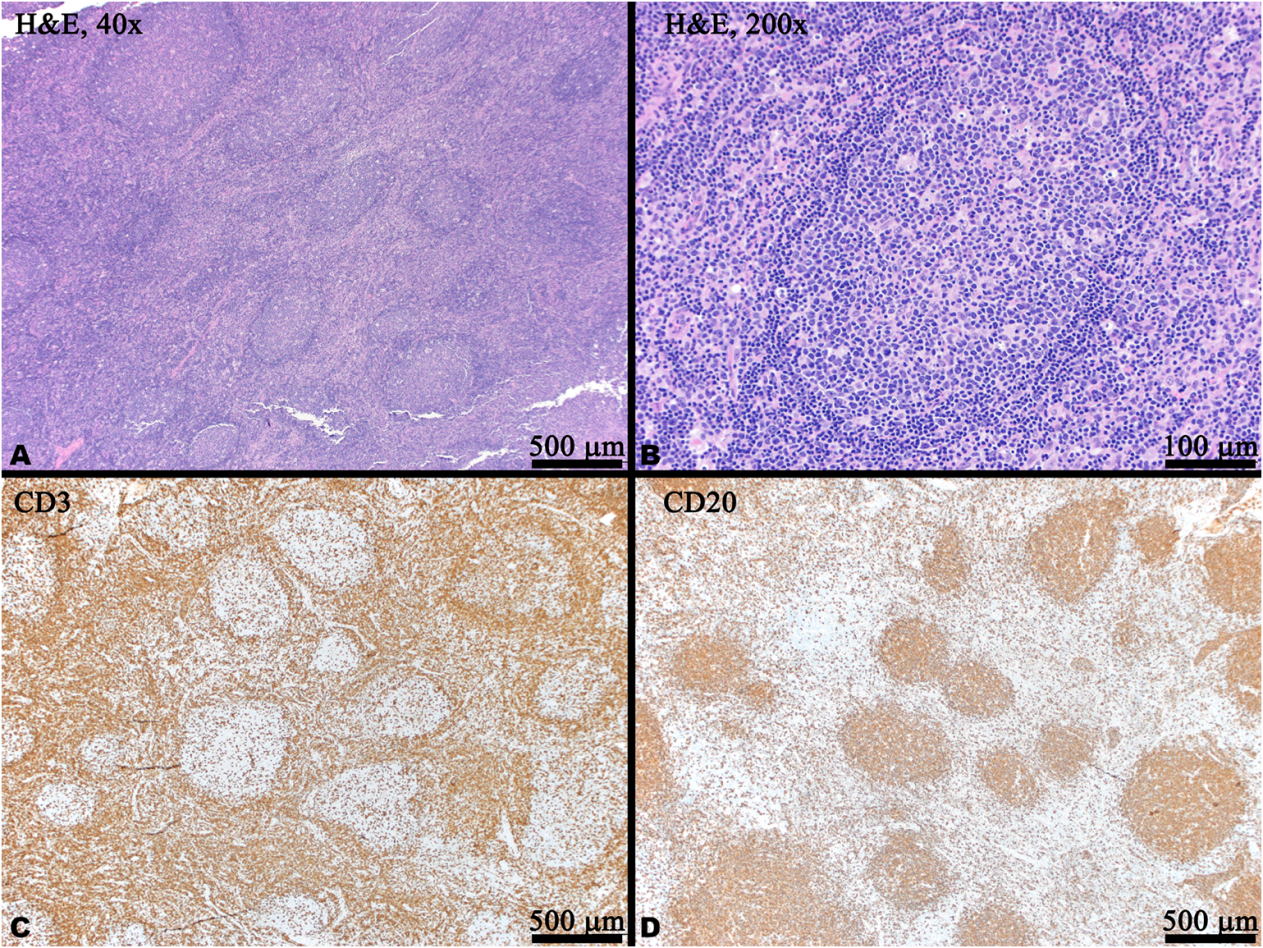
Histology and immunohistochemistry (IHC) of a right cervical lymph node biopsy. **(A)** Low power magnification showing reactive follicular hyperplasia with variably sized and shaped germinal centers with visible mantle zones (H&E). **(B)** Medium power magnification of a reactive germinal center with scattered tangible body macrophage (H&E). **(C)** CD20 IHC labels B cells which are predominantly located within the germinal centers. **(D)** CD3 IHC labels T cells predominantly located in the interfollicular areas and scattered within the germinal centers. In panels **(C, D)**, brown indicates positive staining.

**Figure 3:**
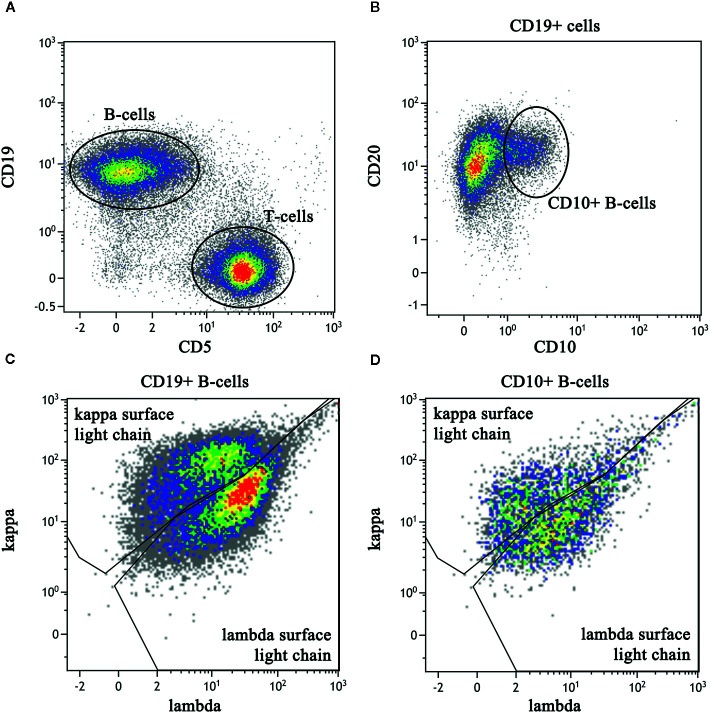
Flow cytometric immunophenotyping dot plots of a right cervical lymph node biopsy. The sample had a viability of 84% as determined by lack of 7-AAD labeling (not shown). **(A)** After exclusion of debris and monocytes, B lineage cells are identified by CD19 labeling (V450) and lineage cells are identified by CD5 (PerCP-Cy5). **(B)** CD19-positive B cells express normal levels of CD20 (APC-H7) with a subset expressing CD10 (APC). **(C)** The entire population of CD19-positive B cells show polytypic expression of kappa (PE) and lambda (FITC) light chain expression, albeit with less than optimal separation kappa and lambda. **(D)** The CD10-positive B cells also demonstrate polytypic light chain expression and are interpreted to represent the reactive germinal centers.

She was referred to a genetic counselor at age 14. Parental consanguinity was noted; the patient’s maternal grandmother and paternal grandmother were sisters. A next generation sequencing (NGS) immunodeficiency panel that included over 200 genes revealed no pathogenic mutations. A repeat excisional lymph node biopsy was performed in the setting of persistent lymphadenopathy; it revealed typical reactive hyperplasia and was negative for EBV RNA by *in situ* hybridization (EBER-ISH) (**Figures 2** and **3**). Whole exome sequencing identified a heterozygous *de novo* pathogenic variant of the *TNFAIP3* gene (c. 1777 C>T in exon 7, p.Q593X); this premature stop codon is presumed to result in A20 haploinsufficiency. In the past year, (age 15), her symptoms have been well controlled with improving lymphadenopathy following escalation of therapy using prednisone, MMF, and two doses of rituximab in addition to the ongoing HCQ and monthly IVIG replacement.

## Discussion

While over 24 unique *TNFAIP3* mutations have been reported that are associated with autoinflammatory and autoimmune disease (reviewed in (7)), to our knowledge this is the first reported case of this particular germline mutation (c. 1777 C>T, p.Q593X) associated with HA20. The specific mutation, C>T at position c.1777 in exon 7, results in a premature stop codon that removes C-terminal Zinc Finger domains. These Zinc Finger domains on exons 4 through 7, when functional, support A20’s ubiquitin ligase activity (3). While biochemical studies have not been performed to test formally whether the mutation described in this report leads to increased TNF-α -associated NF-κB activity, there is high likelihood that this truncating mutation is pathogenic. Molecular and biochemical studies performed by Zhou, et al., who first described HA20, identified five heterozygous truncating mutations either located on an N-terminal domain proximal to the Zinc Finger domains or on the fourth Zinc Finger domain itself that resulted in reduced or no detection of A20 protein and diminished suppressive activity (5). Similar findings showing truncating mutations of most of A20 protein, the same domain as in our patient, or even only the seventh (last) Zinc Finger domain all correlated with HA20 clinical disease as well as diminished A20 expression and function *in vitro* (9). It is important to note that, at the time of publication, the patient described in this study has had testing showing genetic evidence of HA20 without functional biochemical assays, and therefore our diagnosis of HA20 is presumptive and not confirmed.

Whole exome sequencing of this patient and both parents also revealed that this patient’s mutation is a *de novo* mutation, as neither parent harbors the p.Q593X pathogenic variant. A comprehensive review of published cases by Yu and colleagues found only 10 of the 61 reported patients with inflammatory syndromes from HA20 were due to *de novo* mutations – the majority instead have a family history with HA20 inherited in an autosomal dominant fashion (7). Notably, initial reports characterizing extensive family pedigrees with HA20 suggest that while disease penetrance is complete, different patients with the same pathogenic mutation can express a wide range of disease phenotypes (5–8), suggesting that a combination of genetic, epigenetic, and environmental factors likely interact to produce these varied phenotypes. Also notable is that our patient’s *de novo* heterozygous mutation was discovered in the context of confirmed parental consanguinity, a scenario that typically raises suspicion for autosomal recessive conditions.

The initial features of HA20 in this patient included recurrent lymphadenopathy and fevers of unknown origin. In addition to persistently elevated ANA and dsDNA antibodies, by age 11 she also developed more classic features of lupus, including a discoid malar rash and sub-nephrotic range proteinuria. While there is a wide clinical spectrum of illness described in HA20, lupus-like features appear to be rare. A recent review by Yu, et al. that summarized results of 61 patients with HA20 noted that Behcet’s disease was considered as a primary diagnosis in 48% (29 of 61 patients), periodic fever syndromes in 11% (7 patients), while lupus or other connective tissue disease were only first considered in 5% (3 patients, two of whom had prominent mucosal ulcer involvement and the third with an eventual diagnosis of both autoimmune lymphoproliferative syndrome (ALPS) and HA20) (7, 8). Regardless of underlying diagnosis, mucosal ulceration remains a prominent features of HA20; Aeschlimann et al. reported all 16 patients in their cohort of HA20 patients having mucosal ulcers (8). In contrast, mucosal ulcers have not been a feature of our patient’s clinical course at any point in her life. A lack of mucosal ulcers and lupus-like phenotype are both very atypical in comparison to the reported clinical spectrum of HA20, highlighting the need to maintain clinical suspicion of HA20 even in patients without significant Behcet-like illness.

At the age of 12, the patient described in this report developed frequent UTIs, ear infections, and atypical pneumonias, associated with a progressive hypogammaglobulinemia, all of which have improved following monthly intravenous immunoglobulin (IVIG) replacement (**Figure 1**). Notably, rituximab is a CD20 (B-cell) depleting antibody which causes hypogammaglobulinemia in a minority of patients approximately 6 months after treatment, with immunoglobulin recovery observed within the next 6-12 months following last rituximab infusion (10, 11). In contrast, our patient had last received rituximab and mycophenolate at age 10 years, with observed B-cell recovery at age 11 (as assessed by CD19, shown in **Figure 1**) 2 years prior to the onset of hypogammaglobulinemia. At age 14, she began experiencing recurrent infections with associated low immunoglobulin levels prior to the initiation of IVIG (IgG 302, normal 500-1,400 mg/dl; IgA <7, normal 47–249 mg/dl; IgM 57, normal 47–252 mg/dl). However, her absolute B-cell counts at this time remained within normal range (**Figure 1**) and normal germinal center expansion was observed histologically (**Figure 2**), suggesting her hypogammaglobulinemia may be intrinsically due to her disease process rather than incomplete recovery following B-cell depletion treatments. Formally, it remains unclear whether her hypogammaglobulinemia is a direct result of HA20, a consequence of her prior immunomodulatory therapy, or a combination of these factors.

While there are few reports of IgG deficiency in patients with HA20, a recent study indicated that 5 of 16 HA20 patients received IVIG, two of whom had recurrent sinopulmonary infections and a selective IgG subclass deficiency (8), though it is unclear whether these described patients’ need for IVIG was due to the HA20 disease itself or a consequence of immunosuppressive therapies. A recent genetic analysis of patients diagnosed with both chronic variable immune deficiency (CVID) and features of autoimmunity identified a truncating *TNFAIP3* mutation in their cohort (12). The patient in this report has not met clinical criteria for CVID in the context of protective antibody levels detected against both tetanus and diphtheria at age 14 (pneumococcal antibody titers were not investigated) and given the uncertainty of whether her immune suppression was a primary component of her disease *versus* lingering medication effect. However, HA20-associated CVID is intriguing in the context of her persistently low IgG levels, low IgA, and frequent infections, and further studies are needed to more formally interrogate whether HA20 may cause a CVID-like disease with overlapping features of autoimmunity.

Cervical lymphadenopathy remains a prominent feature in this case and is atypical in comparison to other published cases. In a review of patients with HA20, only 5 of 61 patients were reported to have lymphadenopathy (7). Recently, a separate *TNFAIP3* mutation was identified within a cohort of patients with ALPS (13). While ALPS was considered initially, ALPS panels conducted at age 7 and 10 both revealed normal frequencies and numbers of double-negative T-cells. Our patient’s specific p.Q593X A20 mutation has previously been reported in a MALT lymphoma (14) as well as a basal cell carcinoma (15) though both of these mutations were somatic (identified in the tumor cells) whereas our patient’s p.Q593X mutation is germline. While our patient’s lymphadenopathy was identified as typical reactive hyperplasia at age 14 (**Figure 2**), it is unclear whether she is at higher risk for lymphoma. Additionally, independent mouse strains with B-cell specific deletion of A20 have revealed enhanced B-cell proliferation and survival but not spontaneous malignancy (16, 17). These observations suggest that HA20 alone is likely insufficient for the development of lymphoma but that it may promote tumorigenesis in the context of other oncogenes.

## Conclusion

We report a novel mutation of the *TNFAIP3* gene leading to haploinsufficiency of A20 (HA20). While autoinflammatory disease phenotypes are well described in HA20, persistent lymphadenopathy and hypogammaglobulinemia are atypical features. This case highlights the heterogeneity of symptoms that may occur with HA20 and illustrate that HA20 should be considered in patients in whom lupus-like disease features and immunodeficiency overlap. Given the broad role of A20 in suppressing inflammation, immune cell proliferation, and immune responses, further studies elucidating the role of HA20 in tumorigenesis and immunodeficiency should be pursued.

## Data Availability Statement

The original contributions presented in the study are included in the article/supplementary material. Further inquiries can be directed to the corresponding author.

## Author Contributions

ZS drafted the manuscript and prepared figures. SW prepared figures and edited the manuscript. BB oversaw clinical care of the patient and edited the manuscript. All authors contributed to the article and approved the submitted version.

## Conflict of Interest

The authors declare that the research was conducted in the absence of any commercial or financial relationships that could be construed as a potential conflict of interest.
